# Enhancement of Fluid Mixing with U-Shaped Channels on a Rotating Disc

**DOI:** 10.3390/mi11121110

**Published:** 2020-12-15

**Authors:** Chi-Wei Hsu, Po-Tin Shih, Jerry M. Chen

**Affiliations:** Department of Mechanical Engineering, National Chung Hsing University, Taichung 402, Taiwan; ty2jac@yahoo.com.tw (C.-W.H.); quajzmshdg@gmail.com (P.-T.S.)

**Keywords:** centrifugal microfluidics, micromixer, U-shaped channel, Coriolis force, flow visualization, mixing efficiency

## Abstract

In this study, centrifugal microfluidics with a simple geometry of U-shaped structure was designed, fabricated and analyzed to attain rapid and efficient fluid mixing. Visualization experiments together with numerical simulations were carried out to investigate the mixing behavior for the microfluidics with single, double and triple U-shaped structures, where each of the U-structures consisted of four consecutive 90° bends. It is found that the U-shaped structure markedly enhances mixing by transverse secondary flow that is originated from the Coriolis-induced vortices and further intensified by the Dean force generated as the stream turns along the 90° bends. The secondary flow becomes stronger with increasing rotational speed and with more U-shaped structures, hence higher mixing performance. The mixing efficiency measured for the three types of mixers shows a sharp increase with increasing rotational speed in the lower range. As the rotational speed further increases, nearly complete mixing can be achieved at 600 rpm for the triple-U mixer and at 720 rpm for the double-U mixer, while a maximum efficiency level of 83–86% is reached for the single-U mixer. The simulation results that reveal detailed characteristics of the flow and concentration fields are in good agreement with the experiments.

## 1. Introduction

Microfluidics has been widely employed in microtechnology for applications in chemical and biochemical analyses [1,2,3]. Among these applications, micromixing of two or more of fluids often plays a key role in the processes for the analyses [4], such as in chemical selectivity [5], reactors [6,7], extraction [8,9], DNA amplification [10] and DNA microarray [11]. The mixing mechanism in a microscale channel where flow is typically laminar, however, differs from that in a macroscale channel. Without assistance of turbulence, mixing at the microscale occurs mainly via molecular diffusion at the fluid interface. Consequently, it may take a much longer time for complete mixing in microsystems than in macrosystems.

Both active and passive methods have been proposed to enhance mixing at microscale. An extensive review on the recent development of various types and designs of active and passive mixing methods can be found in the articles of Nguyen and Wu [12], Chang and Yang [13] and Ward and Fang [14], and very recently in Raza et al. [15]. The passive method appears to be more appealing than the active one in the applications to chemical and biochemical analyses. This is mainly because in most of active micomixers, it could be more complex and difficult to implement and control the external forcing sources. On the other hand, in order to enhance mixing via secondary flow and possibly chaotic advection, most of passive micromixers are constructed in a rather complex three-dimensional (3D) geometry such as staggered herringbone, serpentine structure and double layers [16,17,18,19,20,21]. Fluid stretching, splitting, folding and recombining are observed in these complex micromixers. However, complex channel structures may hinder the fabrication of the micromixer and its integration with other components. In contrast to 3D structure, Kochmann et al. [22] reported numerical simulations and experiments of high throughput convective micromixing for 2D channels with configurations including T-shaped, U-shaped and tangential elements. They found that the micromixer with U-shaped elements having four successive 90° bends achieved the most effective micromixing mainly due to the vortices generated in the bend flow and the mixing efficiency grew with an increase of Reynolds number up to as high as *Re* ≥ 270. In any case, the complex channel structure tends to raise flow resistance, which leads to increasing the residence time of mixing fluids at the cost of pressure loss. A recent comparative analysis of five types of passive micromixers by Raza et al. [15] shows that a 2D micromixer with split-and-recombination (SAR) Tesla structure outperforms other 2D serpentine and SAR mixers in the intermediate and high Reynolds number ranges (1 < *Re* ≤ 40 and 40 < *Re* ≤ 120, respectively). Nevertheless, a 2D micromixer with Tesla structure, which utilizes the Coanda effect to generate the transverse dispersion resulting in strong cross-channel convection of the mixing, still suffers high pressure drop [15,23,24].

Different from the aforementioned passive mixing techniques with complex channel structure, mixing taking place in centrifuge-driven microchannels of rather simpler geometry promises to be an attractive method with less research [25,26,27]. Centrifugal microfluidics are fabricated onto a compact disc (CD)-based substrate, which only requires a simple, low power motor to spin the disc [28,29,30]. Pumping in such a way is relatively insensitive to the physicochemical properties of the working fluids. Fluid mixing may be performed on the CD-based microfluidics with the advantages of safety as well as low-cost, easy operation, parallel detection, and fast response. Accordingly, applications of centrifugal microfluidic devices to biomedical analysis and point-of-care diagnostics have received increasing attention. Comprehensive reviews on centrifugal microfluidic platforms for biomedical applications has been recently made by Gilmore et al. [31] and Tang et al. [32]. In centrifugal microfluidics, effective sample mixing methods that facilitate chemical or biochemical reaction and reduce the time of the assay is still highly demanded for improving the bioassay devices among with the unit operations of valving, switching, metering, and sequential loading. Notably, the fluids driven by the centrifugal force increases their flow velocity significantly with increasing rotational speed [33]. An increase of rotational speed tends to reduce the residence time of mixing fluids and may result in poor performance without a dedicated design of microfluidics. As the centrifugal microfluidics rotates fast enough, a number of studies have shown that the Coriolis force-induced transverse secondary flow significantly enhances the mixing [30,34,35]. La et al. [35], in a study of a centrifugal serpentine micromixer with relatively long circumferential channels, displayed the significant enhancement of mixing caused by the alternating transverse secondary flows at a high rotational speed of 2000 rpm. Kuo and Jiang [36] compared the CD-based centrifugal micromixers with different curved microchannels and found the square-wave microchannel to have the best mixing efficiency. They further explored the optimal design of the square-wave micromixer in a rotational speed range of 1000–5000 rpm for mixing efficiency as high as 93%. Kuo and Li [37] later studied centrifugal micromixers with three-dimensional square-wave structure for plasma mixing and found that a mixing efficiency of more than 91% could be achieved at 1000 rpm despite a notable decease in the efficiency with a further increase of rotational speed from 1200 to 1600 rpm. Such a higher mixing efficiency is due mainly to the transverse secondary flows and the stirring at the corners of the square-wave channel, which is similar to the mixing enhancement mechanism employed by La et al. [35]. Shamloo et al. [38] recently investigated the Dean flow effect in addition to the already existing Coriolis force in a numerical study of centrifugal micromixer with repeated S-shaped channel for a broad range of rotational speeds of 72–3340 rpm (7.5–350 rad/s). The mixing efficiency computed in their simulations shows a sharp fall with increasing rotational speed in the lower range until it reaches a minimum near 480 rpm. Then the efficiency grows continuously with increasing rotational speed as the transverse secondary flows dominate the diffusion mixing. A continuous growth of mixing efficiency with increasing rotational speed in the range 480–1910 rpm (50–200 rad/s) was also reported by Shamloo et al. [39] in their numerical simulations of centrifugal serpentine micromixers with square-wave structure. The aforementioned studies indicate that dependence of mixing efficiency on rotation speed can vary for centrifugal micromixers with different channel structures and arrangements. Dependence of mixing efficiency on rotational speed is critically important to the design of centrifugal micromixer and needs further investigation. Such a dependence may relate closely to combined effects on the secondary flows caused by the Coriolis and Dean forces, particularly for the microchannel with a simple geometry of 2D structure and yet to be clarified.

The present study aimed to design and fabricate centrifugal microchannels with a simple geometry of U-shaped structure to attain rapid and efficient mixing. The mixers with single, double and triple U-shaped channels fabricated on a disc substrate were investigated using the flow visualization technique. The fluids employed for mixing experiments were ferric chloride and ammonium thiocyanate solutions. The chemical reaction of the two fluids produces a blood-red color that can be employed for flow visualization and quantification of the index of mixing efficiency. Effects of rotational speed on the mixing efficiency and the corresponding flow patterns were carefully examined in the experiments together with numerical simulations. The experimental and numerical studies also examined differences in the flow and concentration fields resulting from counterclockwise and clockwise rotations.

## 2. Experiments

**Figure 1:**
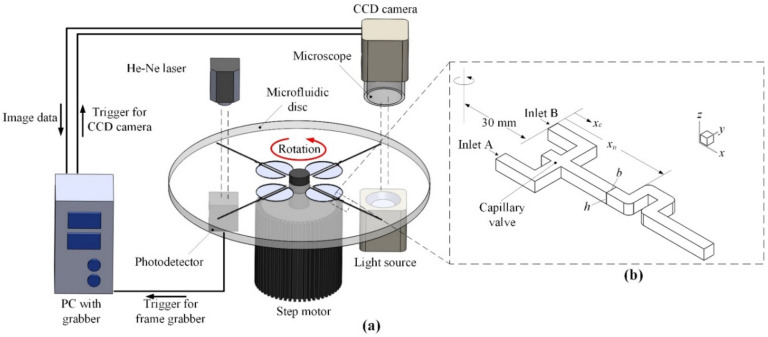
Schematic of the centrifugal U-shaped micromixer: (**a**) experimental arrangement for flow visualization; (**b**) the microchannel geometry and coordinates for single U-shaped structure.

Figure 1 shows the schematic configuration of the centrifugal U-shaped micromixer and the experimental setup for flow visualization of the mixing fluids. The micromixer is composed of two semicircle reservoirs for loading fluids A and B, a T-junction for bringing the fluids into contact, and the U-shaped channel with consecutive four 90° bends. The semicircle reservoirs have a radius of 10 mm with outlets (*x_c_* = 0) located at 30 mm from the center of the disc. A capillary valve with an expansion angle of 90° was fabricated ahead of the T-junction (*x_c_* = 1.3 mm) to stop the flow in the hydrophilic channel before rotation of the disc [28,40]. The burst rotational speed for the present centrifugal micromixers was 360 rpm. It should be noted that the U-shaped channels (beginning from *x_u_* = 3.0 mm) are short and straight with an arc curve at the outer corner while keeping a sharp 90° bend at the inner corner. Such a design enables to rapidly alternate the flow direction for generating strong centrifugal force locally through the consecutive 90° bends. The U-shaped microfluidic structure was manufactured using a micro-CNC machine on a polymethylmethacrylate (PMMA) substrate of 10 cm in diameter and 2 mm in thickness. In addition to the single U-shaped structure shown in Figure 1b, micromixers with double and triple U-shaped structures were fabricated for comparison. All the microfluidic channels have the same cross-section of 300 μm in width (*b*) and 300 μm in depth (*h*). The total radial channel length is 20 mm for all the three types of U-shaped micromixers. The microchannels with double and triple U-structures also begin their first 90° bend from *x_u_* = 3.0 mm and an additional U-shaped element adds a length of 1.2 mm in the *x*-direction. Since the static pressure of the liquid flow in the rotating microchannels is essentially below the atmospheric pressure [33], the structured microfluidics was simply covered with transparent Scotch tape (3040-4PK, 3M, St. Paul, MN, USA) to provide good sealing as well as excellent optical access for flow visualization.

In order to acquire clear images of the mixing phenomenon in the rotating microchannel for the present experiments, the two fluid streams for mixing were ferric chloride (FeCl_3_·6H_2_O) and ammonium thiocyanate (NH_4_SCN) dissolved in deionized (DI) water at the same concentration of 0.2 mol/kg. Ferric chloride solution is pale yellow and ammonium thiocyanate solution is colorless. When these two solutions come into contact, the ferric ions bind with the thiocyanate ions instead of the chloride ions to produce the blood-red color [41]. The intensity of the red color described by the RGB values for each of pixels could represent the amount of fluids that have mixed and reacted. The normalized color intensity was quantified as the index of mixing efficiency between the two fluids.

A schematic diagram of the experimental setup for flow visualization of the mixing phenomenon on a rotating disc is illustrated in Figure 1. The apparatus used for the flow visualization experiments included the rotation platform, the microimage-capturing unit and the light source. The microfluidic disc was spun by a step motor having a speed accuracy of ±0.2%. The microimage-capturing unit was composed of a CCD camera (CV-M71CL, 768 × 576 pixels, 60 frames/s, JAI, Yokohama, Kanagawa, Japan), a frame grabber (Metero-II/CL, Matrox, Dorval, QC, Canada), and a microscope (2× objective) above the disc. To synchronize the image capturing unit with the rotational motion, the CCD camera was triggered through a photo diode (wavelength 320–730 nm). A He-Ne laser (wavelength 633 nm) of 5 mW was used as the triggering light source. A halogen light (MHF-G150LR, Moritex, Saitama, Japan) was placed beneath the disc to produce sufficient light for illuminating the flow field undergoing a rapid rotational motion. In synchronization with the rotational motion, the CCD camera could be triggered to allow one shot of the targeted object on the rotating disc per revolution.

## 3. Numerical Simulation

Numerical simulations were employed to reveal details of the flow patterns and mixing behavior for the fluid streams in the rotating microchannels as shown in Figure 1. All the channels have a total length of 20 mm in the *x*-direction and the same width *b* = 300 μm and depth *h* = 300 μm. Fluids A and B inlet the channels at 30 mm from the center of rotation and then contact at the T-junction (*x_c_* = 1.3 mm) without a capillary valve. The fluids were assumed to have the same constant density as water *ρ* = 1000 kg/m^3^ and the same viscosity *μ* = 0.001 kg/m^2^∙s. The flow field of fluids A and B in the microchannel was computed on the rotating frame. The origin of the computational coordinates is located at the center of the rotating disc. The flow field is governed by the steady, three-dimensional continuity and Navier–Stokes equations:

(1) $$\nabla \cdot \vec{V}=0,$$

(2) $$\rho \vec{V}\cdot \nabla \vec{V}=-\nabla p+\mu \nabla^{2}\vec{V}-2\rho \vec{\Omega }\times \vec{V}-\rho \vec{\Omega }\times \left(\vec{\Omega }\times \vec{r}\right),$$

where $\vec{V}$ is the velocity vector on the reference frame rotating at a constant angular velocity $\vec{\Omega }=\Omega {\vec{e}}_{z}$, *p* is the pressure, and $\vec{r}$ is the position vector. The source terms on the right-hand side of Equation (2) include sequentially the pressure force, the viscous force, the Coriolis force and the centrifugal force. Gravitational effects are ignored. The species concentration for calculating the mixing performance is governed the convection-diffusion equation:

(3) $$\vec{V}\cdot \nabla C=D\nabla^{2}\vec{V},$$

where *D* is the diffusion coefficient, and *C* is the species concentration with *C* = 1 representing only fluid A and *C* = 0 for only fluid B. As the two fluids are completely mixed, *C* = 0.5 The diffusion coefficient for the present simulations, unless otherwise specified, was taken to be at a constant value of 3.0 × 10^−10^ m^2^/s, which was based on the study of Kochman et al. [22].

The finite-volume-based CFD software ANSYS Fluent 15.0 was used to solve Equations (1)–(3) for the two-phase (fluids A and B) flow and concentration fields. The inlet and outlet flow boundary conditions were set at the atmospheric pressure *p* = 0. On the walls of the channels, a no-slip condition was imposed for the Navier-Stokes equations and no-flux condition for the species-concentration equation. Total grids of about 5.6 × 10^5^–6.8 × 10^5^ were employed for the computations.

The mixing efficiency was calculated based on the concentration distribution on a designated cross-sectional plane perpendicular to the fluid stream [18]:

(4) $$\eta_{\mathrm{sim}}=\left(1-\frac{1}{C_{\infty }}\sqrt{\frac{1}{n}\sum_{i=1}^{n}{\left(C_{i}-C_{\infty }\right)}^{2}}\right)\times 100\%,$$

where *C*_∞_ is the completely mixed concentration, *C_i_* is the concentration at a grid *i*, and *n* is the number of grids computed on the cross-sectional plane. The mixing efficiency varies from zero (no mixing at all) to unity (complete mixing) is generally a function of the streamwise position. For the present numerical simulations, the mixing efficiency was evaluated on Section 5 as indicated in Figure 1, which was located at the immediate exit of the most downstream U-shaped element (*x_c_* = 4.2, 5.4 and 6.6 mm for single, double and triple U-shaped mixers, respectively).

## 4. Results and Discussion

### 4.1. Simulation Results

**Figure 2:**
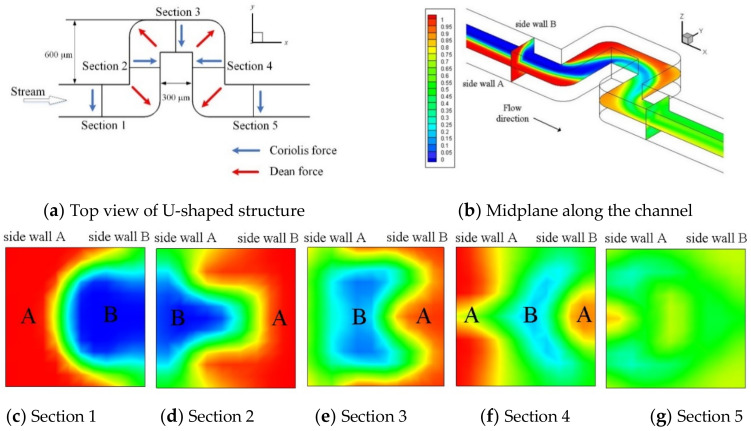
Top view of the U-shaped channel and concentration distribution of fluids A and B for the single-U mixer rotating at Ω = 600 rpm (counter-clockwise; ccw): (**a**) top view with detailed dimensions and cross-sections selected for examination as well as the directions of Coriolis and Dean forces acting on the fluids; (**b**) distribution on mid-plane along the channel; (**c**–**g**) on cross-sections 1–5 normal to the fluid stream as indicated in (**b**).

Figure 2 shows the top view of the single-U mixer with directions of the body forces and the simulation result of concentration for the mixer rotating at 600 rpm counterclockwise (ccw). The fluids in the rotating U-shaped channel can experience three types of body forces, namely the centrifugal force *f_ω_* generated through the system rotation, the Coriolis force *f_C_*, and the Dean force *f_D_*, which is the centrifugal force locally generated through the consecutive 90° bends [36,38]. The magnitude of these three forces may be estimated as follows [42]:

(5) $$f_{\omega }=\rho r\Omega^{2},$$

(6) $$f_{C}=2\rho \Omega U,$$

(7) $$f_{D}=\frac{\rho U^{2}}{R},$$

where *r* is the distance of the fluid to the center of rotation, *U* is the stream velocity in the channel, and *R* is the radius of the stream bended in the channel. The directions of Coriolis and Dean forces vary rapidly along the U-shaped channel as indicted in Figure 2a, where the stream is driven by system’s centrifugal force under a counterclockwise rotation. Notably the Coriolis and Dean forces alternate not only in direction but also in position between Sections 2 and 4. The concentration distribution (with values between 0 and 1) at the midplanes is shown in Figure 2b. At the inlets, red color represents fluid A of species concentration 1.0, and blue color fluid B of concentration 0. As the two fluids fully mix, the color turns green representing a concentration of 0.5. It can be seen that the Coriolis-induced transverse secondary flow begins to influence the interface in the straight channel region, moving fluid A toward upper and lower walls and fluid B toward sidewall A to form a “C-shaped” interface in Section 1 at *x_c_* = 2.7 mm. Subsequently, the 90° bends appear to stir the fluids by flipping fluids A and B in Section 2. This can be seen first in Section 2 changing from AB to BA and flipping again in Section 3. Then in Section 4 where fluid A splits to sandwich fluid B forming ABA. In addition to flipping, interface folding also becomes more significant as the flow moves to Section 4. After flowing through these bends, the two fluids are well mixed in Section 5 (*x_c_* = 4.2 mm). Similar patterns of flipping, splitting, folding and stirring were also observed in the study by La et al. [35] but with a much longer circumferential channel length of 5.2 mm (17 times the channel width) at a much higher rotational speed Ω = 2000 rpm. It should be noted that there is no Dean force generated in a long circumferential channel (*R* → ∞) [38], in which the secondary flow is essentially induced by the Coriolis force.

**Figure 3:**
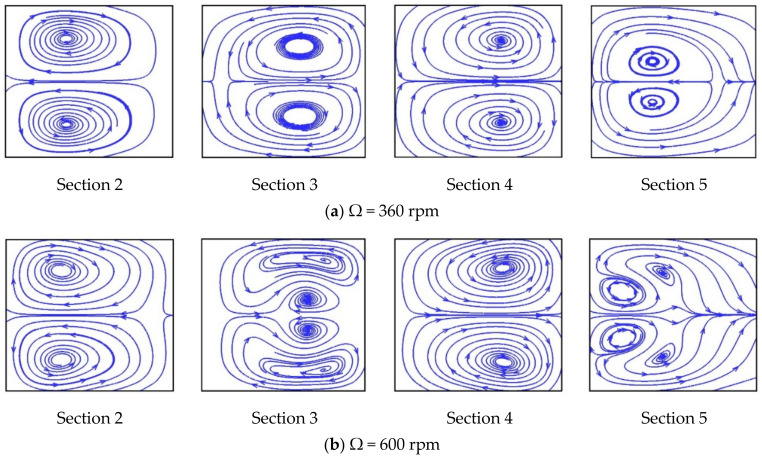
Two-dimensional streamlines on cross-sections 2–5 as indicated in Figure 2a for the single U-shaped mixer: (**a**) rotating at Ω = 360 rpm (ccw); (**b**) rotating at Ω = 600 rpm (ccw).

**Figure 4:**
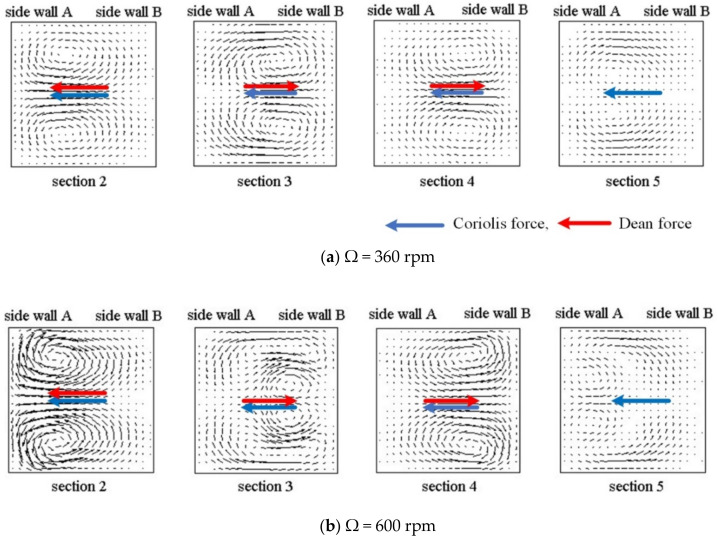
Velocity vector field with the directions of Coriolis and Dean forces on cross sections 2–5 as indicated in Figure 2a for the single U-shaped mixer: (**a**) rotating at Ω = 360 rpm (ccw); (**b**) rotating at Ω = 600 rpm (ccw).

Figure 3 and Figure 4 show streamlines and velocity vectors, respectively, together with directions of the Coriolis and Dean forces on cross-sections 2–5 for the single U-mixer rotating at Ω = 360 and 600 rpm ccw. There are at least a pair of symmetric, counter-rotating vortices induced by the Coriolis and Dean forces on all the cross-sections for both two rotational speeds. At Ω = 360, these secondary vortices reverse their directions of rotation after the flow makes each of the 90° turns and then a saddle point can be found in Section 5. The reversed rotation of the secondary vortices after the 90° turns indicates effects of the Dean force, which as shown in Figure 4a alternates its direction pointing from side walls B toward A (B→A) to A→B on Sections 3 and 4. In the meantime, the Coriolis force remains unchanged pointing from side walls B toward A (B→A) through the four 90° turns. At a higher rotational speed Ω = 600 rpm, one more pair of symmetric, counter-rotating vortices appear in Sections 3 and 5, indicating that the flow is significantly affected not only by the Coriolis force but also by the Dean forces in the transverse direction during the 90° turns. The significant effect of the Dean force can be seen in Figure 4b displaying larger transverse velocities on Sections 3 and 4 as compared those at the lower rotational speed of Ω = 360 rpm. The strong transverse secondary flow initiated by the Coriolis force and then intensified by the Dean force through the 90° bends markedly enhances the fluid mixing as shown in Figure 2. 

The coupled Coriolis–Dean effects may be examined from the ratio *γ_c_* of the two forces given in Equations (6) and (7) as [42]:

(8) $$\gamma_{c}=\frac{f_{D}}{f_{C}}=\frac{U}{2R\Omega },$$

where the stream velocity *U* is a function of rotational speed Ω. Then *U* can be taken to be the mean velocity *U_m_* and expressed as:

(9) $$U_{m}\propto \Omega^{k},$$

with *k* representing the power-relation exponent. For a straight and round channel (*R* → ∞) on a rotating disc, the power-relation exponent *k* = 2 approximately [26]. For the single-U mixer employed in the present study, *k* ≈ 1.84 in the lower rotational speed range (Ω ≤ 480 rpm) and the exponent becomes smaller *k* ≈ 1.41 in the higher range (Ω ≥ 600 rpm). Taking the case of Ω = 600 rpm as an example, with *U_m_* = 0.38 m/s and an average radius *R* ≈ (2^1/2^)*b* = 424 µm, the ratio of the Dean force to the Coriolis force is approximated to be *γ_c_* ≈ 7.1. This means that the Dean force dominates the Coriolis force in generating the secondary flow as the stream turns along the consecutive 90° bends, and the Dean–Coriolis force ratio increases with increasing rotational speed. 

In addition, it is worth looking into the effect of the system’s centrifugal force on secondary flows, which play a major role in enhancing mixing of the present study, as compared to those of the Coriolis force and the Dean force. For a radial straight microchannel of rectangular cross-section, the ratio of the system’s centrifugal force to the Coriolis force may be scaled as [30,34]:

(10) $$\frac{f_{\omega }}{f_{C}}=\frac{r\Omega }{2U}=\frac{4\mu }{\rho b^{2}\Omega },$$

where the mean stream velocity *U_m_* instead of the maximum stream velocity *U_max_* is used to scale the Coriolis force and a characteristic scaling *f_C_* ∝ Ω^2^ is invoked. As already mentioned, the mean stream velocity *U_m_* depends on Ω, and the exponent *k* in Equation (9) tends to decrease in the higher rotational speed range for the single-U mixer. It should be noted that in Equation (10), the Coriolis force is perpendicular to the radial direction of the system’s centrifugal force. The transverse secondary flow accompanied by the streamwise vorticity in the radial straight channel is mainly due to the Coriolis force, while the system’s centrifugal force contributes to drive the stream flow along the channel [43,44]. When examining the effect of the system’s centrifugal force on secondary flows in the circumferential channel section (orthogonal to the radial direction), great care is required because *f_ω_* is parallel to *f_C_*. This effect may be understood based on vorticity consideration by rewriting Equation (2) for a constant angular velocity Ω as [43,44]:

(11) $$\rho \vec{V}\cdot \nabla \vec{V}=-\nabla p^{*}+\mu \nabla^{2}\vec{V}-2\rho \vec{\Omega }\times \vec{V},$$

where the centrifugal force is incorporated into the modified pressure p^*^ given by

(12) $$p^{*}=p-\frac{1}{2}\rho \Omega^{2}(x^{2}+y^{2}).$$

The vorticity equation for incompressible flow can then be obtained by taking the curl of Equation (11) as [45,46]:

(13) $$(\vec{V}\cdot \nabla )\vec{\omega }=(\vec{\omega }\cdot \nabla )\vec{V}+\nu \nabla^{2}\vec{\omega }-2\nabla \times (\vec{\Omega }\times \vec{V}),$$

where $\vec{\omega }=\nabla \times \vec{V}$ is the vorticity and ν is the kinematic viscosity. The three source terms on the right-hand side of Equation (13) represent the vortex stretching, the viscous diffusion of vorticity and the Coriolis-force production of vorticity, respectively. Note that the modified pressure does not appear in the vorticity equation since the curl of the gradient of any scalar is zero. This indicates that the system’s centrifugal force does not ‘directly’ contribute to generation of vorticity in the interior of the channel [46,47]. However, this does not mean that the system’s centrifugal force plays no role in generation of streamwise vorticity in a channel section that is orthogonal to the radial direction. The system’s centrifugal force, nevertheless, has important influences in helping vorticity generation in microchannel flow through change of the boundary geometry (e.g., a sudden expansion or a curved bend) and variation in pressure gradient along the channel’s solid surface [47]. For the flow in the U-shaped channel structure with a very short circumferential section, the streamwise vorticities that lead to producing and intensifying the transverse secondary flow are generated mainly from stretching and turning of vortex lines through the consecutive 90° bends of the channel as well as from the Coriolis force. The streamwise vorticities generated when the flow turning along the consecutive bends are, in the present study, accounted for as the Dean–Coriolis force effect, which is indeed originated from the flow driven by the system’s centrifugal force.

**Figure 5:**
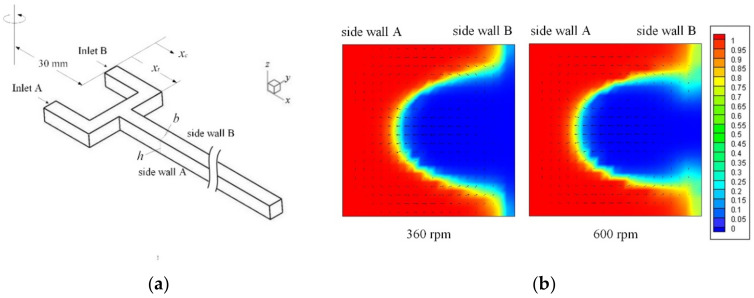
Centrifugal T-type micromixer and cross-sectional concentration distribution: (**a**) schematic of the T-type micromixer showing the channel geometry and coordinates; (**b**) concentration distributions and velocity vectors at *x_c_* = 4.2 mm for Ω = 360 and 600 rpm (ccw).

Effects of the coupled Dean–Coriolis forces can also be revealed through comparison of centrifugal mixing characteristics between the U-shaped mixer and a T-type mixer. Figure 5a illustrates schematic of the centrifugal T-type micromixer employed for the present simulations. The T-type micromixer with fluid inlets located at 30 mm from the center of the disc is simply a straight channel having the same cross-section (*b* = *h* = 300 μm), radial length (exit at *x_c_* = 20 mm) and junction position (*x_j_* = 1.3 mm) as the U-mixer. Figure 5b displays the cross-sectional concentration distributions and velocity vectors computed at *x_c_* = 4.2, which is the same *x*-position as Section 5 of the U-mixer, for two rotational speeds Ω = 360 ad 600 rpm (ccw). A C-shaped interface of the fluids can be clearly seen in the cross-section for both rotational speeds. In the T-mixer, the transverse secondary flow is induced essentially by the Coriolis force alone [30] since there is no Dean force in the straight channel (*R* → ∞). It can be seen from the transverse velocity vectors that the secondary flow drives the fluids from side walls B toward A (B→A) in the middle of the cross-section neighboring *z* = 0, where the maximum radial velocity occurs, and A→B near the top and bottom walls. When comparing these two cross-sectional distributions, the C-shaped interface for the higher rotational speed of 600 rpm appears to be a bit wider and lighter near the corners on side wall B than that of 360 rpm. This indicates a slightly better mixing for the higher rotational speed due to a stronger Coriolis-induced secondary flow that compensates for the shorter residence time of the mixing fluid stream. 

**Figure 6:**
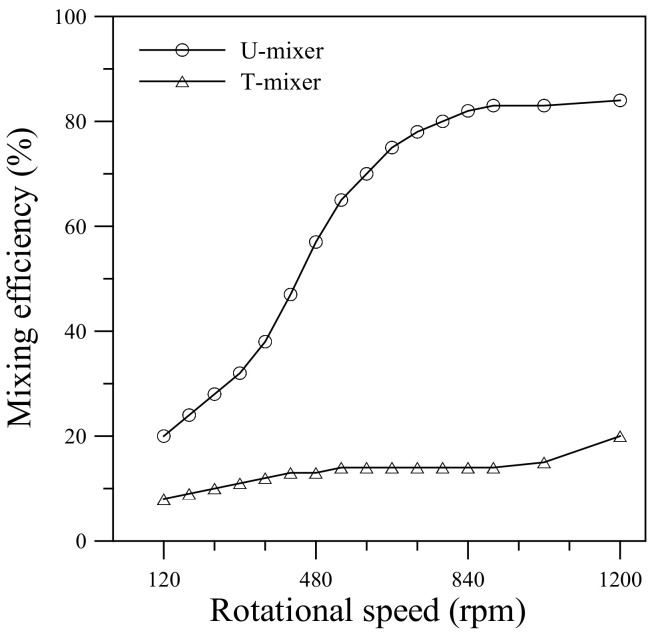
Comparison of mixing efficiency for the single U-shaped mixer and T-type mixer rotating at different speeds from 120 to 1200 rpm (ccw).

Figure 6 compares mixing efficiency of the single U-shaped mixer with the T-type mixer at different rotational speeds ranging from 120 to 1200 rpm (ccw). The mixing efficiency was calculated using Equation (4) based on the cross-sectional concentration distribution in Section 5 (*x_c_* = 4.2 mm) for the U-mixer and at the same *x*-position (*x_c_* = 4.2 mm) for the T-mixer. It can be seen that mixing efficiency for the U-mixer increases rapidly with rotational speed, from 20% at Ω = 120 rpm to 82% at Ω = 840 rpm. Beyond Ω = 840 rpm, the mixing efficiency appears to level off around 83–84%. The flat level efficiency in the higher speed range is due mainly to the high flow velocity that increases rapidly with rotational speed. The mean velocities at the U-mixer exit were found to be 0.67 m/s for Ω = 900 rpm and 1.0 m/s for Ω = 1200 rpm, corresponding to Reynolds numbers *Re* = 200 and 300. The rapidly increasing velocity reduces the fluid residence time and appears to offset the growing influence of transverse secondary flow. It is worth mentioning that the simulations with different diffusion coefficients (1.0 × 10^-12^ and 3.0 × 10^-9^ m^2^/s) also exhibit a similar trend of mixing efficiency variation with rotational speed as in Figure 6 for the same U-shaped mixer, but give a slightly higher efficiency (by an amount of 5–6%) for the larger diffusion coefficient and slightly lower efficiency (by an amount of 4–5%) for the smaller diffusion coefficient in the level-off range Ω = 900–1200 rpm. In other words, changes in mixing efficiency contributed from the diffusion coefficient variation by an order of magnitude are limited. This indicates that advection plays the key role in enhancing fluid mixing for the present centrifugal U-shaped microchannel.

In Figure 6, the T-mixer also shows an overall growth in mixing efficiency with increasing rotational speed due mainly to the Coriolis-induced secondary flow. However, the fluids flowing through the straight channel of the T-mixer without U-turns have a much lower mixing efficiency (8–20%) than that observed in the U-mixer. The comparison in Figure 6 points out that Coriolis-induced vortices alone may not help mixing the fluids so much in a rotating microchannel. It is necessary to have the Dean flow generated during rapid turns of fluid stream along the consecutive bends to strengthen the transverse secondary flow for further enhancement of the mixing. The simulation results also indicate that the centrifugal U-mixer that enables effective assistance of fluid mixing within a short channel length is very suitable for use in CD-based microfluidic systems. Moreover, an additional U-shaped structure can be expected to further enhance the fluid mixing by maintaining a large value of *γ_c_* for a longer channel length.

### 4.2. Experimental Results and Comparison

**Figure 7:**
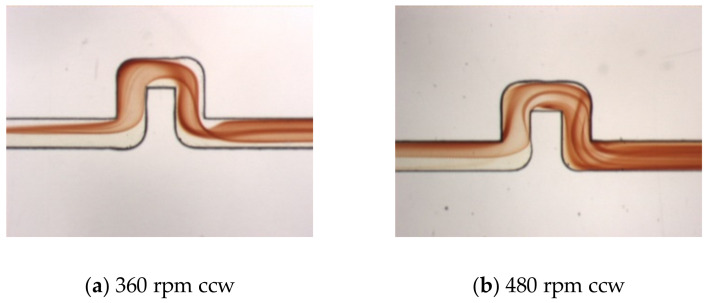

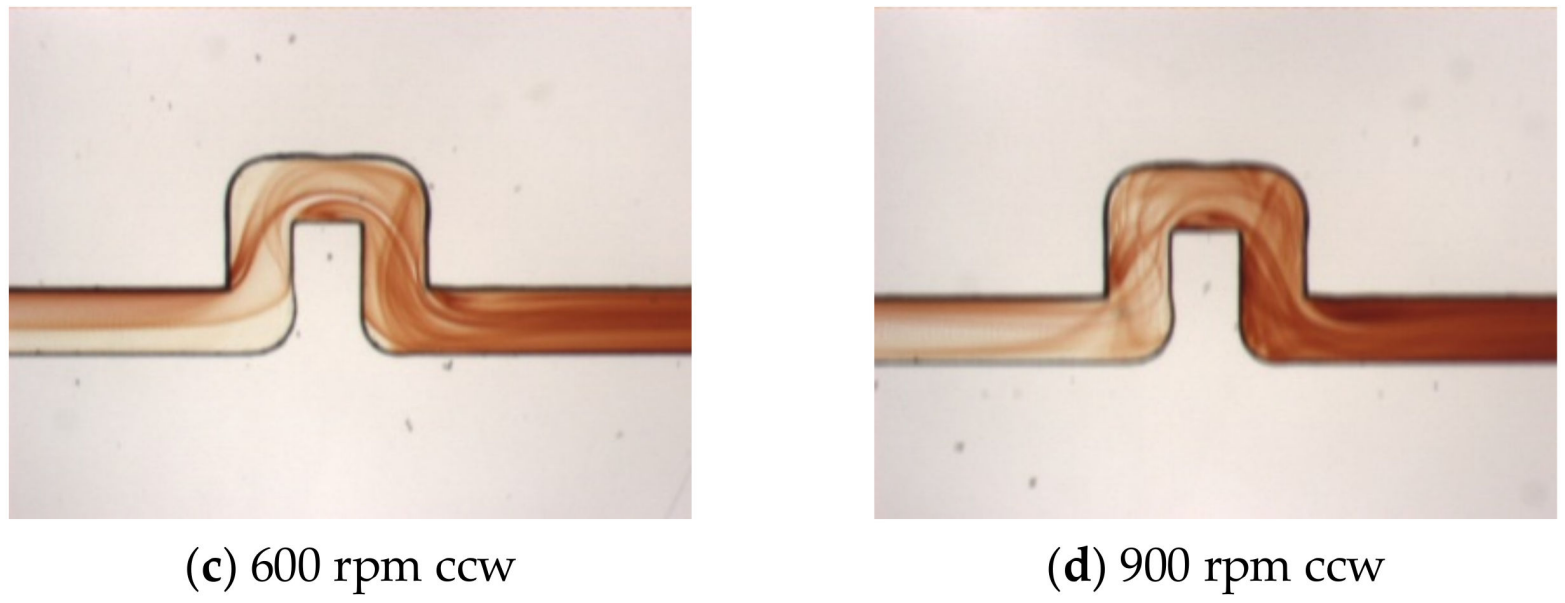
(**a**–**d**) Top view visualization of the mixed fluids for the single U-shaped mixer rotating from Ω = 360 to 900 rpm (ccw), where the flow enters from the left.

Figure 7 shows flow visualization of top view images for the single-U mixer rotating at different speeds from 360 to 900 rpm (ccw). The flow enters the U-shaped channel from the left. At a lower speed of 360 rpm, the colored fluid appears on the upper side of the entrance region due to the effect of Coriolis force. The colored area indicates the mixed interface where the two testing fluids contact and react. The Coriolis force, in this counterclockwise rotating case, points downward on the top view images. As a result, the colored area in the entrance region appears in the upper half of the image, reflecting the C-shaped interface observed on Section 1 of Figure 2c. The mixed area becomes wider but lighter, signifying stretching of the interface, as flow turns along the first and second 90° bends. Then along the third and fourth bends, the mixed interface clearly shows a twisting shape accompanied by the stretching phenomenon. At the exit of the U-shaped structure, the mixed area increases largely than at the entrance. As the speed increases to 480 rpm, the mixed area is enlarged in the entrance region where stretching and twisting of the interface take place slightly earlier than at the lower speed. Arriving at the fourth 90° bend, the colored lines’ entanglement implies folding and twisting of the interface. Thereafter the testing fluids mix well at the exit of the U-shaped structure. As the speed further increases to 600 and 900 rpm, folding and twisting of the interface occurs even earlier. As a result, the colored area covers nearly the whole downstream of the U-structure. It can be seen that the mixing performance is significantly enhanced at the exit for Ω = 900 rpm. Note that, at the higher speeds, the colored area appears to spread from the upper wall toward the down side but with lighter color. This is a result of large ratio of Dean–Coriolis forces that tends to widen the interface but the faster flow velocity, particularly in the neighborhood of the channel centerline, allows less time for the fluid interface to diffuse.

**Figure 8:**
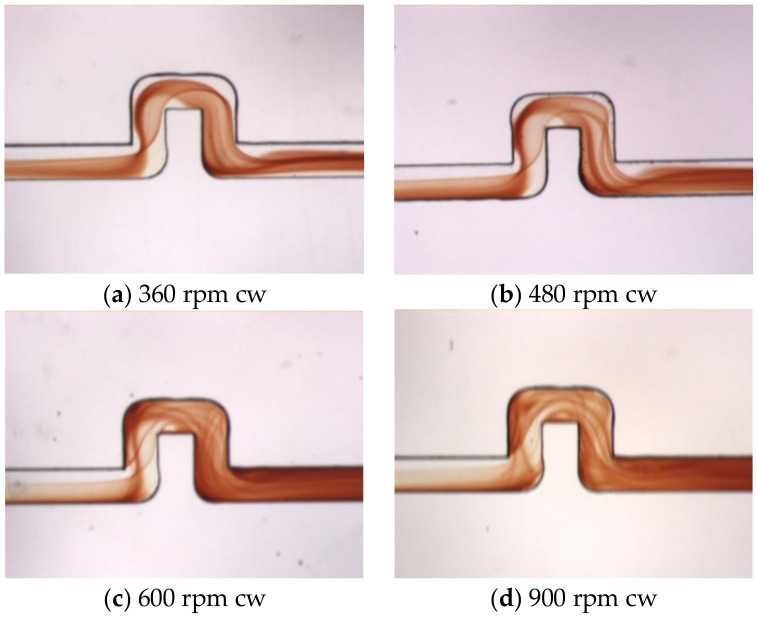
(**a**–**d**) Top view visualization of the mixed fluids for the single U-shaped mixer rotating from Ω = 360 to 900 rpm (clockwise; cw), where the flow enters from the left.

Under the effects of the Coriolis force, the present U-shaped mixer is not fully symmetric between the clockwise and counterclockwise rotations. Figure 8 shows flow visualization of top view images for the same U-mixer of Figure 7 but rotating clockwise (cw) at speeds from 360 to 900 rpm. The Coriolis force now points upward on the top view images for the flow in the *x*-direction. Therefore the colored interface develops on the lower side of the image in the entrance region and becomes wider but lighter as the rotational speed increases. At lower speeds Ω = 360 and 480 rpm, it is observed that the interface turns around the second and third 90° bends without touching the upper wall of the radial channel between Sections 2 and 4 because of the upward Coriolis force, unlike those shown in Figure 7a,b for the counterclockwise rotation. At higher speeds Ω = 600 and 900 rpm, the interface broadens and covers most of the channel after Section 2 due mainly to the stronger Dean force produced as the stream with a higher velocity turns along the 90° bends. It is also observed that at these higher velocities, stretching, folding and twisting of the fluid interface appear to display a lot more entangled lines and surfaces. As a result, the mixing at the exit becomes much better than at the lower speeds. 

**Figure 9:**
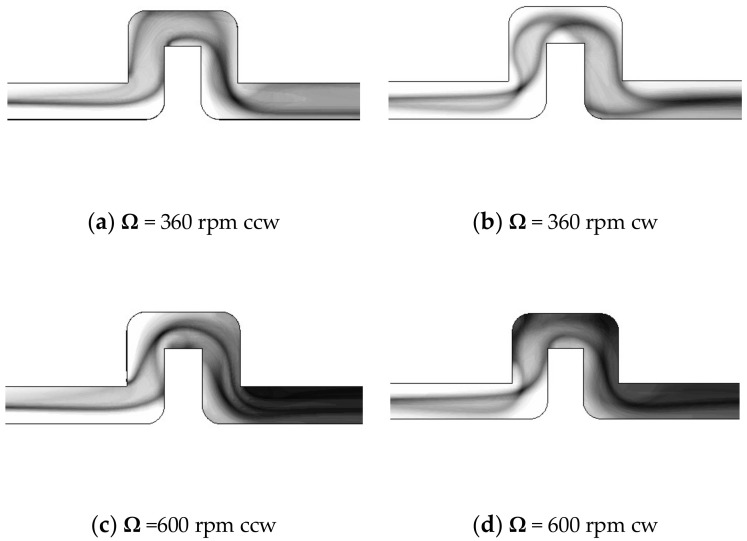
(**a**–**d**) Top view images of fluid mixing obtained from numerical simulations for the single U-shaped mixer at Ω = 360 and 600 rpm undergoing both counterclockwise and clockwise rotations, where the flow enters from the left.

Figure 9 shows the top view images of numerical simulations displaying the fluid mixing phenomenon for Ω = 360 and 600 rpm undergoing both counterclockwise and clockwise rotations. Each of these simulation images are composed of the species concentration distributions on the *x*-*y* plane in the upper half of the channel (from *z* = 0 to 150 μm). The concentration distributions are shown here in gray scale with black representing the complete mixing (*C* = 0.5) and white for unmixed fluids (*C* = 0 or 1.0). The composite distribution images were made for comparison with the visualization images of Figure 7 and Figure 8, which accumulated the entire color changes through the depth of the channel. For both lower and higher rotational speeds, the simulation images clearly demonstrate the interface shape that is affected by the Coriolis force and the Dean force as the stream turns along the 90° bends. The Coriolis effects caused by counterclockwise rotation can be distinct from those by clockwise rotation. For the higher rotational speed Ω = 600 rpm, the twisted and overlapped lines are largely increased to exhibit a darker surface, particularly near the exit of the U-shaped structure, indicating a higher mixing performance. The simulation images of Figure 9 are consistent with and closely resemble those observed in the experiments having the same speeds and directions of rotation. 

**Figure 10:**
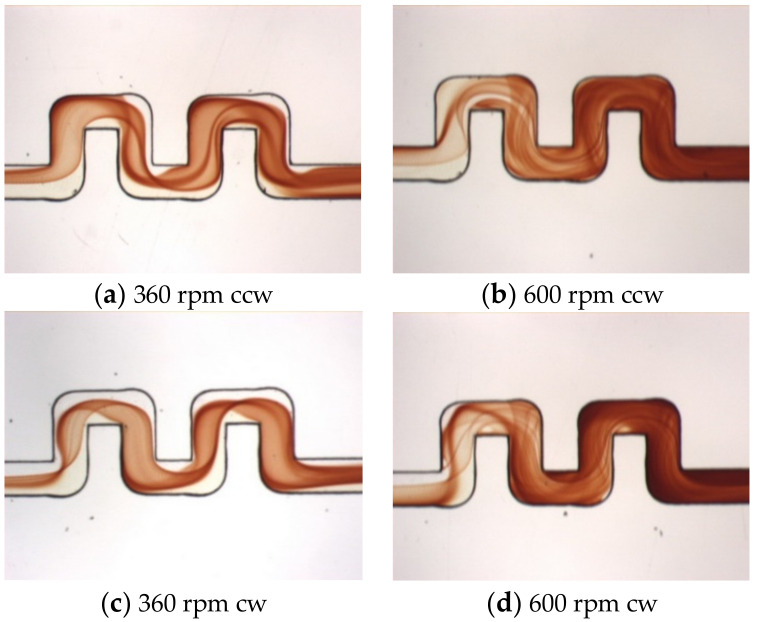
(**a**–**d**) Top view visualization of the mixed fluids for the double-U mixer undergoing counterclockwise (ccw) and clockwise (cw) rotations at 360 and 600 rpm, where the flow enters from the left.

Figure 10 shows flow visualization of the double-U mixer at rotational speeds of 360 and 600 rpm. Both clockwise and counterclockwise rotations are presented for comparison. At the lower speed Ω = 360 rpm, with one more U-structure, it can be seen that the colored lines and surfaces representing the mixed fluid interface become more stretching and twisting in the second U-structure. For the higher speed Ω = 600 rpm, the colored lines and surfaces are largely entangled. For both lower and higher rotational speeds, the mixed area at the exit of the second U-shaped structure becomes darker than that observed in the single U-shaped structure. The second U-structure apparently further enhances the mixing through the intensified transverse secondary flow caused by the Coriolis force and the Dean force in particular during the four more 90° turns. In the clockwise rotation cases, the Coriolis force that points upward causes the mixed interface to move toward the lower half of the entrance channel. The stretching, twisting and folding phenomenon of the interface appears to be similar to that in the clockwise single-U mixer but larger in strength. The mixed area at the exit of the second U-structure for the higher rotational speed Ω = 600 rpm is observed to be darker than that for the single U-shaped channel.

**Figure 11:**
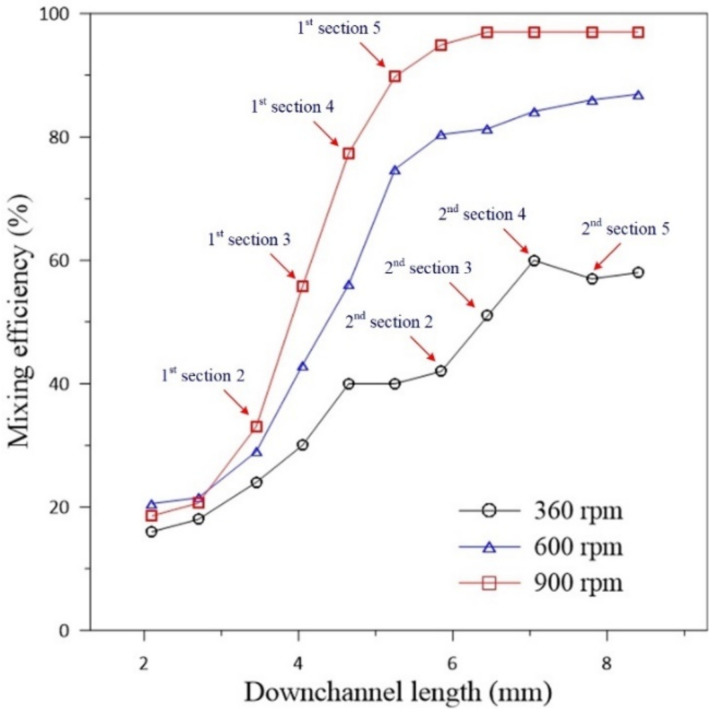
Variations of simulation mixing efficiency in downstream channel distance for the double-U mixer undergoing counterclockwise rotations at 360, 600 and 900 rpm with indication of downstream positions for the cross-sections of the first and second U-structures.

Figure 11 presents the simulation results for the variations of mixing efficiency with the downstream channel distance beginning from the T-junction for the double-U mixer at Ω = 360, 600 and 900 rpm (ccw). At the lower speed Ω = 360 rpm, the mixing efficiency increases significantly at Sections 2–4 of the first U-structure and is further enhanced in at Sections 2–4 of the second U-structure. There is a small decline between Sections 4 and 5 of the second U-structure, which may be due to the fact that the rapidly changing flow directions are not perpendicular to the cross-sections during the turns, causing the concentration collected on the cross-section to have a small variation with the flow angle. At the higher speeds Ω = 600 and 900 rpm, the mixing efficiency dramatically grows at Sections 2–5 of the first U-structure. Then the efficiency still maintains a significant growth but with a less steep slope in the second U-structure. The dramatic growth followed by a significant rise in mixing efficiency is due largely to the strong Dean flow caused by the high Dean-Coriolis-force ratio (*γ_c_* ≈ 6.2 and 7.4 for Ω = 600 and 900, respectively).

**Figure 12:**
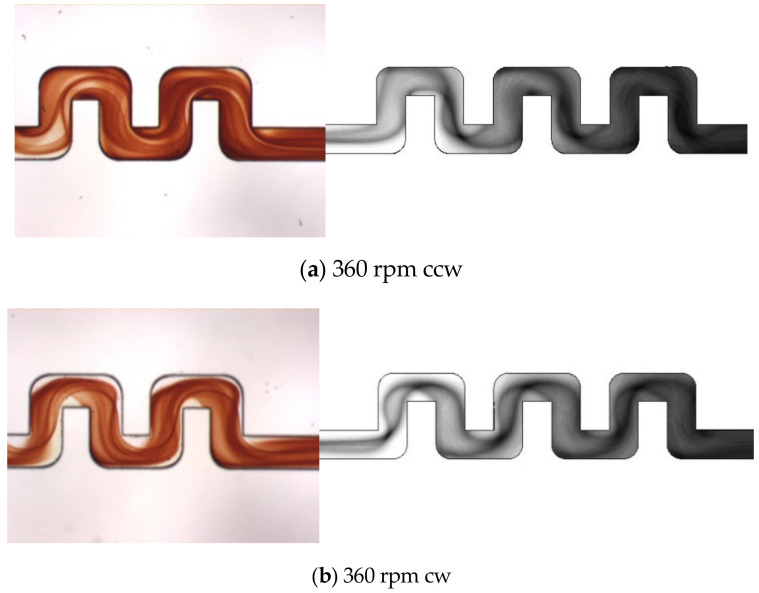
Top view images of fluid mixing from visualization experiments (**left**, where only the second and third U-structures are displayed) and numerical simulations (**right**) for the triple-U mixer at Ω = 360 rpm: (**a**) counterclockwise rotation; (**b**) clockwise rotation. The flow enters from the left.

Figure 12 shows top-view mixing images of flow visualization and numerical simulations of the triple-U mixer at a rotational speed of 360 rpm for counterclockwise and clockwise rotations. For the flow visualization images, only flow in the second and third U-structures are displayed because the visualization was designated to target the exit of the third U-structure for quantifying mixing efficiency. It can be observed that colored surfaces of the mixed-fluid interface in the second and third U-structures are even more stretching and twisting than those in double-U mixer at the same speed, indicating more stirring. It can also be seen from the visualization images that the counterclockwise rotation mixing appears to better the clockwise case. This could be due to the combined effects of Dean and Coriolis forces, which are toward more opposite directions between Sections 2 and 4 for the counterclockwise case, resulting in larger stirring of the mixing fluids than the clockwise case. The simulation images, displaying the flow along the whole three U-structures, clearly demonstrate the difference in interface shape between the counterclockwise and clockwise rotation cases as those shown in the flow visualization. The twisted and overlapped interface representing the mixed fluids become more entangled and darker in the second and third U-structures, indicating that mixing enhancement can be achieved with more U-structures. 

**Figure 13:**
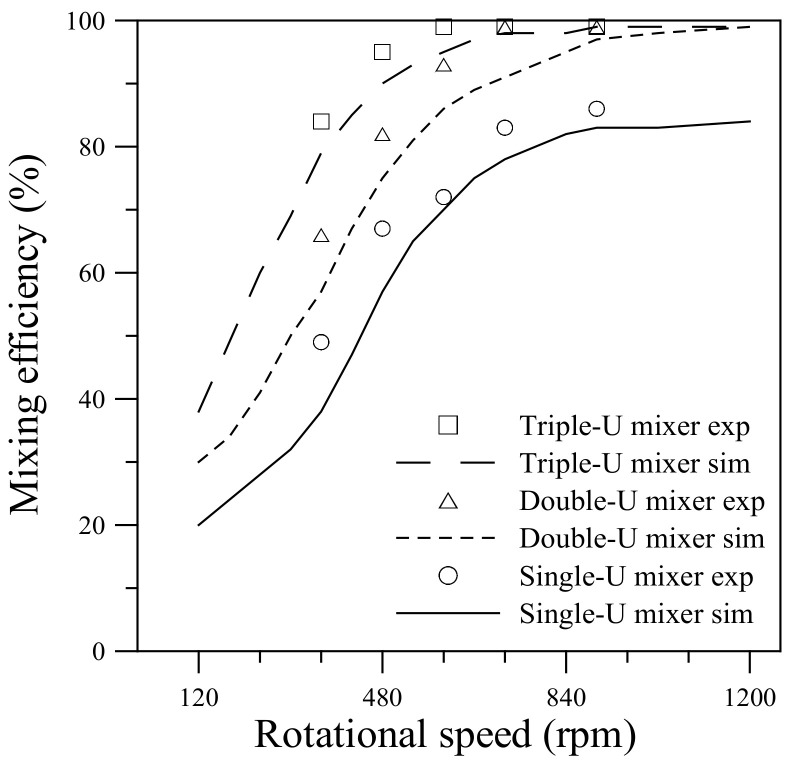
Comparison of mixing efficiency between experiments and simulations for the single-, double- and triple-U mixers undergoing counterclockwise rotation in the range 120–1200 rpm (360–900 rpm for experiments).

**Figure 14:**
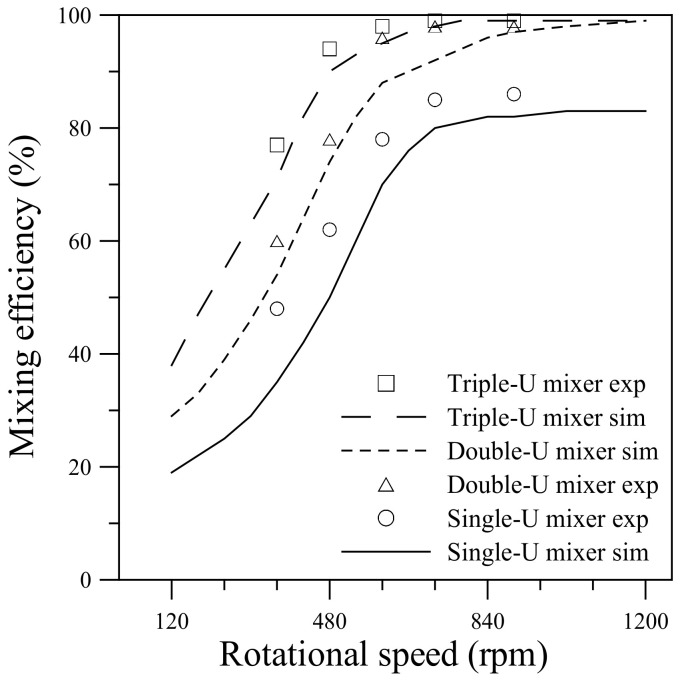
Comparison of mixing efficiency between experiments and simulations for the single-, double- and triple-U mixers undergoing clockwise rotation in the range 120–1200 rpm (360–900 rpm for experiments).

Figure 13 compares the mixing efficiencies measured from the visualization experiments with those obtained from the numerical simulations for the single-, double- and triple-U mixers undergoing counterclockwise rotation at different speeds. A similar comparison for the three mixers undergoing clockwise rotation is presented in Figure 14. Quantification of the mixing performance in the present experiments is based on the concentrations measured in the imaged area located at the exit of the U-shaped structure. The imaged area was approximately 450 μm × 400 μm with its left side located at *x_c_* = 4.2, 5.4 and 6.6 mm for the single-, double- and triple-U mixer, respectively. The mixing efficiency computed from the pixel intensity of averaged RGB values in the imaged area is given by [16]:

(14) $$\eta_{\exp}=\frac{I-I_{\min}}{I_{\max}-I_{\min}}\text{×}100\%.$$

The maximum red intensity *I*_max_ is observed in a fully mixed image and the minimum intensity *I*_min_ is observed in an image of DI water. The technique of quantifying mixing efficiency from concentration measurements was employed previously by Chen et al. [48]. For all the three U-mixers, the mixing efficiency measured from the visualization experiments increases sharply with increasing rotational speed in the lower range, Ω = 360 to 600 rpm for the single- and double-U mixers and an even lower range of Ω = 360 to 480 rpm for the triple-U mixer. As the speed further increases to 720 and 900 rpm, the efficiency gradually levels off for the single-U mixer. Nearly complete mixing is attained at Ω = 720 rpm for the double-U mixer and at an even lower speed of Ω = 600 rpm for the triple-U mixer. This increasing trend is observed in both counterclockwise and clockwise cases and is well predicted by the simulations with a constant diffusion coefficient of 3 × 10^−10^ m^2^/s. 

For the single-U mixer undergoing counterclockwise rotation, the measured mixing efficiency increases from 48% at Ω = 360 rpm to 72% at Ω = 600 rpm and reaches 86% at Ω = 900 rpm. The clockwise rotation case is slightly lower than the counterclockwise rotation by about 5% at Ω = 480 rpm. But at Ω = 600 rpm, the clockwise rotation jumps to lead the counterclockwise rotation by approximately 6% and then approaches a maximum of 86% as the speed further increases. For the double-U mixer, the measured mixing efficiency is apparently higher than that for the single-U mixer by an amount of 10–20%. The efficiency increases from 60–65% at Ω = 360 rpm to reach nearly 99% at Ω = 720 and 900 rpm for both clockwise and counterclockwise cases. This is consistent with the observation of Figure 10. An additional U-shaped structure further enhances the mixing through the intensified transverse secondary flow caused by the Coriolis and Dean forces with four more 90° turns of the stream. In the meantime, the mean velocity at the exit of the double-U mixer is found to be slower than that of the single-U mixer by 13–17%, which leads to an increase of fluid residence time giving help to the mixing. The mean velocity was estimated from the visualization images beginning from the burst of flow until the fluid reservoirs become empty. It was found that the mean velocity reduced from 0.16 m/s for the single-U mixer to 0.14 m/s for the double-U mixer at the burst rotational speed Ω = 360 rpm, and from 0.74 to 0.64 m/s at the highest speed Ω = 900 rpm. For the triple-U mixer under counterclockwise rotation, the measured mixing efficiency was found to be higher than 80% even at a lower speed Ω = 360 rpm, subsequently grows to 95% at Ω = 480 rpm and then reaches a nearly complete mixing value of 99% at Ω = 600 rpm and beyond. In the clockwise rotation case, efficiency for the triple-U mixer also significantly better than those for single- and double-U mixers, attaining about 95% at Ω = 480 and 600 rpm, and rising to near complete mixing at Ω = 720 and 900 rpm. For a lower speed of 360 rpm, the efficiency appears to be 7% lower than that of counterclockwise rotation at the same speed, as observed in Figure 12. The simulations that cover a rotational range of 120–1200 rpm show in good agreement with measurements. Overall, the simulation efficiencies appear slightly lower than the measured values with an average difference of about 4%. At Ω = 900, the simulation mixing efficiency approaches 97% for the double-U mixer and 99% for the triple-U mixer. With one or two more U-structures, the simulation mean velocity was also found to be slower becoming 0.59 and 0.53 m/s at Ω = 900 rpm for the double- and triple-U mixers, respectively. Moreover, the simulations show no discernible difference in mixing efficiency between clockwise and counterclockwise cases, for which the difference in mean velocity is very small (~1%) at the same rotational speed.

## 5. Conclusions

Centrifugal microfluidics with a simple geometry of U-shaped channel was investigated for achievement of rapid and efficient mixing of the fluids. Three types of microfluidics, namely microchannels with single-, double- and triple-U structures, were examined in the present study. Experimentally, the testing fluids of ferric chloride and ammonium thiocyanate solutions were employed for flow visualization of the mixing. The blood-red color produced as the two fluids in contact and reaction provides clear images for qualitative interface visualization as well as quantitative evaluation of mixing efficiency. Numerical simulations were also carried out to reveal detailed characteristics of the flow and concentration fields. It is found that the fluid mixing is remarkably enhanced by the transverse secondary flow. The secondary flow originated from the Coriolis-induced vortices due to the channel flow on a rotating disc is further intensified by the Dean force generated as the stream turns along each of the 90° bends of the U-shaped structures. A scaling parameter *γ_c_*, similar to the one used by Zhang et al. [42], is introduced to examine effects of the Dean force as compared to the Coriolis force. Stretching, twisting and folding of mixing interface caused by the transverse secondary flow were observed in flow visualization and closely resembled in numerical simulation as well. It is also found that the secondary flow becomes stronger with increasing rotational speed and with more U-shaped structures, resulting in a larger area of mixed fluids and hence higher mixing performance. The mixing efficiency measured for the three types of U-shaped mixers shows a sharp increase with increasing rotational speed in the lower range Ω ≤ 600 rpm. As the speed further increases, nearly complete mixing can be achieved at Ω = 600 for the triple-U mixer and at Ω = 720 rpm for the double-U mixer, while a maximum level between 83 and 86% is reached for the single-U mixer at Ω = 720 and 900 rpm. The variation of simulated mixing efficiency with rotational speed in a wider range (120–1200 rpm) agrees well with the measurements. Moreover, both the simulation and measurement results show no discernible difference in mixing efficiency between clockwise and counter-clockwise cases. The centrifugal U-shaped micromixers presented in this study enabling to assist fluid mixing effectively within a short channel length are especially suitable for use in CD-based microfluidic systems.
